# “We were caught sleeping”: formulation of national policy to decentralise management of drug-resistant tuberculosis in South Africa – a qualitative policy analysis study

**DOI:** 10.1136/bmjopen-2025-115476

**Published:** 2026-09-28

**Authors:** Mosa Moshabela, Lindy Dickson, Waasila Jassat, Helen Cox, Karina Kielmann, Marian Loveday, John Black, Koleka Mlisana, Mark P. Nicol, Alison D. Grant

**Affiliations:** 1 Africa Health Research Institute, School of Laboratory Medicine and Medical Sciences, College of Health Sciences, University of KwaZulu-Natal, Durban, South Africa; 2 School of Nursing and Public Health, University of KwaZulu-Natal, Durban, South Africa; 3 Department of Pathology, Division of Medical Microbiology, University of Cape Town, Cape Town, South Africa; 4 School of Biomedical Sciences, University of Western Australia, Perth, Australia, USA; 5 School of Public Health, University of the Western Cape, Bellville, South Africa; 6 School of Public Health, University of the Witwatersrand, Johannesburg, South Africa; 7 Institute of Infectious Disease and Molecular Medicine and Wellcome Centre for Infectious Disease Research in Africa, University of Cape Town, Cape Town, South Africa; 8 Burnet Institute, Melbourne, Victoria, Australia; 9 Institute of Global Health and Development, Queen Margaret University, Edinburgh, UK; 10 HIV and other Infectious Diseases Research Unit, South African Medical Research Council, Cape Town, South Africa; 11 Centre for the AIDS Programme of Research in South Africa (CAPRISA), Nelson R Mandela School of Medicine, University of KwaZulu-Natal, Durban, South Africa; 12 Department of Infectious Diseases, Livingstone Hospital, Port Elizabeth, South Africa; 13 Division of Infectious Diseases, University of Cape Town, Cape Town, South Africa; 14 School of Laboratory Medicine and Medical Sciences, College of Health Sciences, University of KwaZulu-Natal, Durban, KwaZulu-Natal, South Africa; 15 National Health Laboratory Service, Johannesburg, South Africa; 16 Marshall Centre, School of Biomedical Sciences, The University of Western Australia, Perth, Western Australia, Australia; 17 TB Centre, Faculty of Infectious and Tropical Diseases, London School of Hygiene & Tropical Medicine, London, UK

**Keywords:** Tuberculosis, Health policy, Public health, South Africa, Quality of health care

## Abstract

**Abstract:**

**Objective:**

In response to growing numbers of people with drug-resistant tuberculosis (DR-TB), South Africa was, in 2011, among the first countries to launch a national policy on the decentralisation of DR-TB management, preceding normative guidance from the World Health Organization. The objective was to understand the process of formulation of the South African policy on DR-TB service decentralisation.

**Design:**

Qualitative policy analysis. We reviewed policy-related documents to create a timeline, then conducted in-depth interviews with key individuals who had influence over or insights into the policy formulation process. We used Kingdon’s policy analysis framework to examine the convergence of problem, policy and politics streams in agenda-setting to create the window of opportunity for policy adoption.

**Setting:**

South Africa.

**Participants:**

Policy makers, implementers and researchers in three South African provinces and at national level, and international DR-TB experts.

**Results:**

International publicity concerning the extensively drug-resistant TB outbreak in Tugela Ferry positioned DR-TB as a public health crisis. The health system lacked capacity to match hospital bed supply in specialised hospitals to the high number of people with DR-TB. Reports of detention against will, victimisation and stigmatisation, and failure to uphold person-centred approaches, sparked a human rights outcry. Tensions between actors favouring ‘control-based’ approaches with emphasis on centralisation and control of patient treatment and resources, and those favouring ‘access-based’ approaches that emphasised decentralised care led to a tipping point in the policy process. Centralised, specialised DR-TB approaches were perceived to protect quality of care, but these compromised person-centred care and human rights, and restricted access to DR-TB treatment. Policy actors used lessons from pilot studies of DR-TB decentralisation and HIV programmes to overcome policy stagnation.

**Conclusions:**

Policy makers should monitor demand and supply of specialised services and support evidence-informed policy change before health system pressures make reform unavoidable.

**STRENGTHS AND LIMITATIONS OF THIS STUDY**

- We captured the views of key actors critical to the process of formulating the policy to decentralise drug-resistant tuberculosis (TB) care in South Africa.
- Key informant interviews offered rich and diverse perspectives on the policy process, highlighting the non-linear nature of policy formulation.
- Key informants’ recall of the events and timeline of the policy process is subjective and thus may present an imperfect picture.
- We captured primarily the experience of policymakers and advocates and may have underestimated the influence of those driving policy from within lower tiers of the national TB programme.

## Introduction

“Those who cannot remember the past are condemned to repeat it” [attributed to George Santayana].

Following the declaration of tuberculosis (TB) as a global emergency in 1993, the World Health Organization (WHO) launched the ‘DOTS’ (Directly Observed Treatment, Short-Course) strategy,^1^ with key components including sputum smear microscopy for TB diagnosis and a dependable supply of high-quality drugs for drug-sensitive TB.^1^ The DOTS strategy was endorsed as cost-effective by the World Bank in 1993^2 3^ and widely adopted. Drug-resistant TB (DR-TB) subsequently emerged as a major threat to global public health,^1–3^ particularly in low- and middle-income countries,^4 5^ exacerbated by pervasive inequitable access to diagnostics and treatment due to historical failures of international policy and related funding strategies.^1 2 5^ An outbreak of DR-TB in New York City in the late 1980s prompted a national action plan in the USA including universal mycobacterial culture and drug-susceptibility testing, access to second-line anti-tuberculous drugs and strict infection control measures.^1 6 7^

WHO’s DOTS strategy considered individualised diagnostic and treatment algorithms for DR-TB too costly^4^ and a distraction from effective delivery of care for drug-sensitive TB.^8 9^ However, in response to the increasing incidence of DR-TB, and mounting evidence of successful pilot DR-TB treatment projects in low- and middle-income countries, the DOTS-Plus strategy was introduced in 2000.^10^ When Peru identified an outbreak of DR-TB in 1995, the national government and non-governmental organisations (NGOs) adapted the New York City individualised management strategy to develop a successful large-scale, ambulatory community-based care model.^11^ This contrasted with DOTS-Plus, which, under the direction of the Green Light Committee, supported controlled provision of second-line drugs to people with DR-TB^12–14^ that was largely centralised, defined as limited to certain health facilities or special units selected for this purpose.^15^

South Africa, a middle-income country with a high prevalence of HIV, TB and DR-TB, adopted the DOTS-Plus strategy in 2001. Accordingly, care for DR-TB was highly centralised, with inpatient treatment and care provided solely by specialised DR-TB centres or teams for the duration of the intensive phase of therapy or until culture or smear conversion. In 2006, South Africa came under the international spotlight when high case-fatality rates were reported during an outbreak of extensively drug-resistant TB (XDR-TB) in Tugela Ferry, a rural area in KwaZulu-Natal province.^16 17^ In response, deliberations on how to manage the crisis led to the beginning of a shift in national policy, resulting in a policy to decentralise services for DR-TB in 2011; at this time there was no normative guidance from WHO concerning decentralisation of DR-TB care. Decentralised care is defined as care provided in the community where the patient lives, by non-specialised workers, but can include an initial period of hospitalisation.^15^

Variability in implementation resulted in models of decentralisation which differed across South African provinces.^18–23^ The policy events and processes that led to the launch of the South African DR-TB decentralisation policy have not been fully described; we therefore conducted an in-depth analysis of the policy formulation process. The objectives of this study were to (1) identify the triggers and motivations behind decisions to introduce the policy; (2) explore the trajectory of events leading to its formulation; (3) establish the confluence of tensions and opportunities leading to the ‘tipping point’ of policy formulation and (4) explain the outcomes of the DR-TB decentralisation policy process in South Africa.

## Methods

### Study design

We undertook a qualitative policy analysis study to understand the process of formulation of South Africa’s policy on decentralisation of care for people with DR-TB. We examined DR-TB decentralisation in three provinces of South Africa, selecting provinces with high TB prevalence but with diversity of geographic, socioeconomic and political characteristics.^24 25^ The three provinces had contrasting approaches to decentralisation of DR-TB care^21^: Western Cape and KwaZulu-Natal piloted decentralisation strategies prior to the adoption of the national policy whereas Eastern Cape did not.^21 26^

To investigate the processes leading to development of a national policy to decentralise DR-TB care in South Africa, we needed a framework concerned with agenda setting and policy formulation, two stages of the policy implementation cycle heuristic framework.^27^ Accordingly, our analysis was guided by Kingdon’s multiple streams theory of agenda-setting, which has been used to analyse how global health issues get onto the policy agenda, including in African countries.^28 29^ Kingdon’s model^30 31^ considers three streams: the problem stream (public policy issues requiring intervention), the policy stream (analyses and discussion of the problem, and possible solutions) and the politics stream (the influence of government, national opinion and interest groups); these three streams are considered to flow relatively independently until circumstances (such as a ‘crisis’ in which the problem is acutely manifest) make them converge and result in an agenda-setting opportunity, or ‘policy window’ that leads to policy change.^29–31^

### Research team and reflexivity

Data were collected by MM and WJ, both South Africans with previous experience delivering clinical care in South Africa and research experience on DR-TB, policy and health systems, working part-time on the project. There was no previous formal relationship between interviewers and participants although some South African participants were publicly known as experts. Participants were aware of the researchers’ names and the research aims. The interviewers reported no bias or assumptions regarding the research. Some of the authors of this paper (not including MM and WJ who did the interviews) had roles in policy formulation.

### Data collection: document review

We started by reviewing national-level, provincial-level and metropolitan-level documents from the three study provinces to establish a timeline of key events and decisions leading up to the policy adoption. Policy documents, draft policies, guidelines, clinical presentations and meeting minutes were systematically compiled. Our electronic search architecture used databases including PubMed, Web of Science and Google Scholar as well as grey literature searches using Google, from January 2004 to December 2016. Search terms included combinations of (‘drug-resistant tuberculosis’ OR ‘MDR-TB’ OR ‘XDR-TB’) AND (‘decentralisation’ OR ‘decentralised’ OR ‘community-based care’). We restricted results to English-language policy-relevant text, excluding clinical drug trial registries. We reviewed the texts to extract key dates, milestones and operational shifts and synthesised these data into a timeline documenting critical events and the historical trajectory of DR-TB decentralisation. This timeline served as a foundation to anchor and contextualise our key informant findings.

### Data collection: key informant interviews

Key informant interviews were conducted by MM and WJ in 2016–2017. People were eligible as key informants if they had been actively engaged in the formulation of, advocacy for or high-level administration of DR-TB policy guidelines in South Africa between 2006 and 2011. Potential participants were identified via an institutional stakeholder mapping exercise, which included reviewing historical policy task team allocations, acknowledgements within official national Department of Health documents, and peer-referrals from established national DR-TB advisory panels. Purposeful maximum variation sampling was then applied to select potential key informants. Additional key informants were identified during the study based on emerging findings and accounts of participants regarding influential actors during the agenda setting process. Participants were invited by email; none declined to take part or withdrew. Probe questions were piloted on local role players with knowledge of DR-TB to familiarise interviewers with the flow of questions.

In-depth interviews were conducted face-to-face in workplaces or hotels, except those with two experts outside South Africa, conducted over Skype. Questions were not provided in advance. Only the researchers and the participant were present during interviews. Interviews lasted 50–70 min depending on the extent of information shared and were not repeated. The interview guide (online supplemental material) covered policy agenda triggers; barriers and facilitators of the policy process; role of actors in the policy process; and factors related to implementation of the policy. All interviews were conducted in English and audio-recorded. Field notes were not taken. Interviews stopped when thematic saturation was reached. This was assessed operationally during the final three interviews during which no new historical incidents, structural barriers or novel relational dynamics between the stream components were reported by participants, indicating informational redundancy.

### Data management

Data were managed with NVivo. Interview data were kept in locked cupboards accessible only to the transcriber (SLR, included in acknowledgements) who transcribed verbatim. Transcripts were allocated a study identifier to ensure anonymity; recordings were then deleted. Transcripts were stored securely on a password-protected local data storage drive providing perimeter protection, managed access controls and replication to an off-site facility. Transcripts were not returned to participants for comment.

### Data analysis

We used a framework analysis approach informed by Kingdon’s Multiple Streams Framework^30^ to analyse the primary qualitative data from key informant interview transcripts. This framework provided our *a priori* operational codebook, sorting data from transcripts into three macro-themes: problem stream, policy stream and politics stream. Within these macro-themes, open thematic coding was executed inductively to generate descriptive subcodes as shown in figure 1 (eg, ‘mandatory admissions’, ‘DOTS-Plus strategy’, ‘resistance vs innovation’). Initial coding was done by MM, then cross-checked across transcripts, using a data matrix aligned with the themes and subthemes (figure 1) to assess consistency, and finally cross-verified at workshops with all authors where discrepancies in coding or interpretation were resolved by consensus.

**Figure 1:**
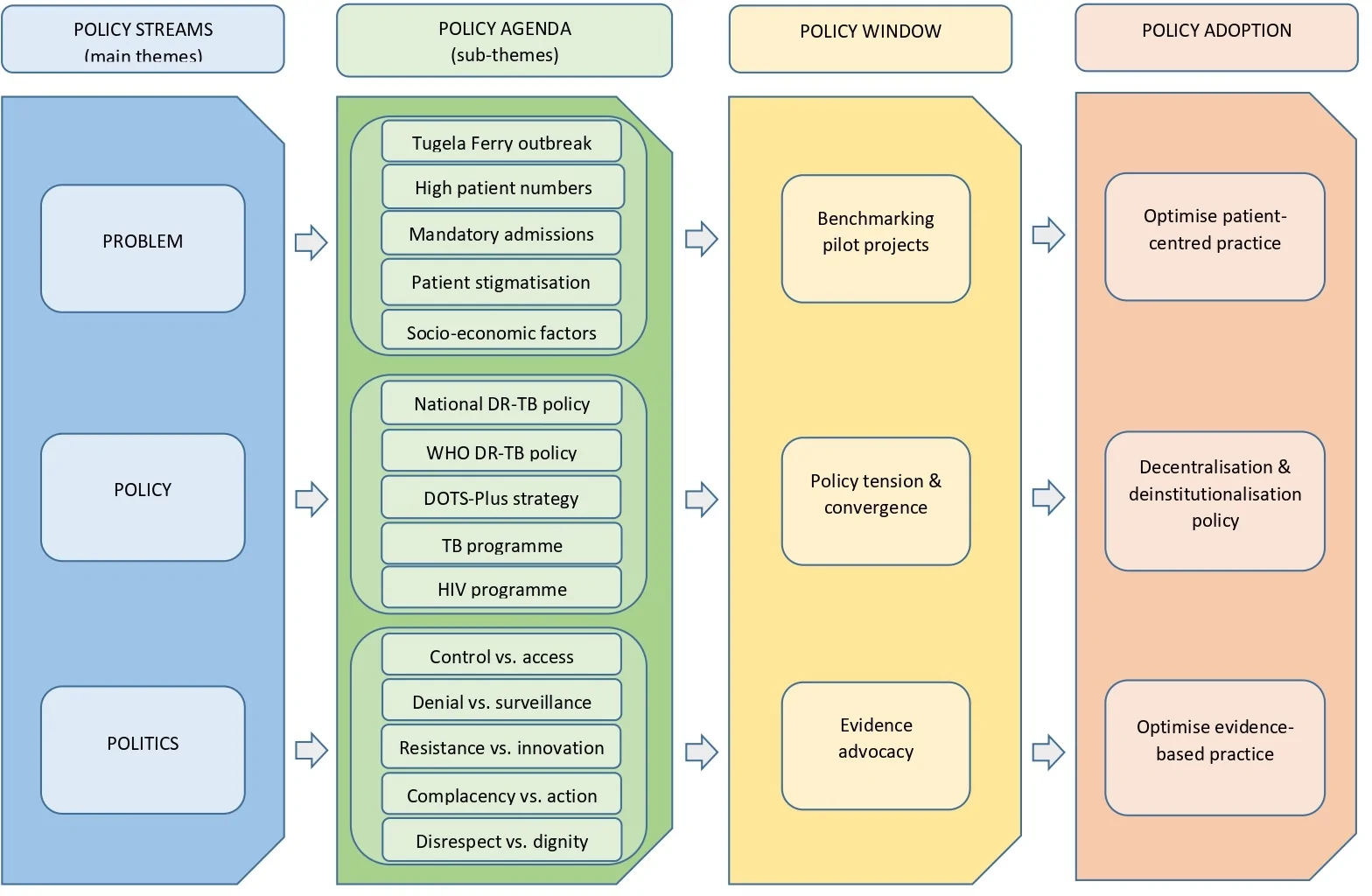
Main themes and subthemes, and how these came together in the development of national policy for decentralisation of drug-resistant tuberculosis (DR-TB) in South Africa. DOTS, Directly Observed Treatment, Short-Course.

### Patient and public involvement

This study involved neither patients nor members of the public but was informed by media reports, published literature and government reports drawn from routine data. Participants included DR-TB experts, policymakers, programme implementers, health activists and advocates. Study findings were disseminated at national DR-TB meetings and presented to programme implementers from all nine South African provinces.

## Results

We interviewed 30 actors (16 female) categorised as senior governmental DR-TB programme implementers (hereafter ‘public sector implementers’, n=10); national DR-TB policy makers (‘policy-makers’, n=5); DR-TB researchers (‘researchers’, n=5); non-governmental actors involved in DR-TB (‘NGO actors’, n=5) and global DR-TB actors who participated in DR-TB policy formulation in South Africa (‘global actors’, n=5).

### Document review: chronology of events

The policy timeline for DR-TB (figure 2) indicates that South Africa was already reporting on the number of people with DR-TB in 1996.^32^ The prevailing view of experts was that DR-TB was primarily a consequence of poor DS-TB treatment, and therefore effective control of DS-TB would control DR-TB^23 33^; accordingly, DS-TB treatment was prioritised, with a re-treatment regimen for people previously treated, in line with the WHO DOTS strategy and South African DR-TB guidelines, introduced in 1997. However, despite guidelines, sputum culture and sensitivity testing were substantially under-requested.^34^ In 2001, the WHO DOTS-Plus strategy^13^ was introduced with a standardised second-line treatment regimen for DR-TB. The 2000 and 2001 South African national DR-TB guidelines recommended hospital admission for several months until the person was sputum-culture negative, in keeping with the WHO DOTS-Plus strategy.^13 23^ The institutional focus of these documents was structural restriction; they omitted any operational guidance for community ambulatory management or patient autonomy, effectively treating non-adherence as a regulatory violation rather than a failure of access to care. Problems with the standardised second-line treatment regimen, including poor outcomes due to limited drug options and unacceptable side effects of some drugs, did not inspire confidence in the DR-TB programme.^14 35^ In 2004, the South African government declared TB a national emergency driven largely by rising prevalence of untreated HIV disease, and adopted a TB crisis plan. In 2005, the news media in South Africa reported the first cases of a ‘superbug’, a strain of *Mycobacterium tuberculosis* with extensive drug resistance, later termed XDR-TB.^36^ The policy change process can be traced back to events related to this ‘superbug’, soon after the 2006 Lancet publication describing the XDR-TB outbreak in Tugela Ferry, a rural town in KwaZulu-Natal province.^16^

**Figure 2:**
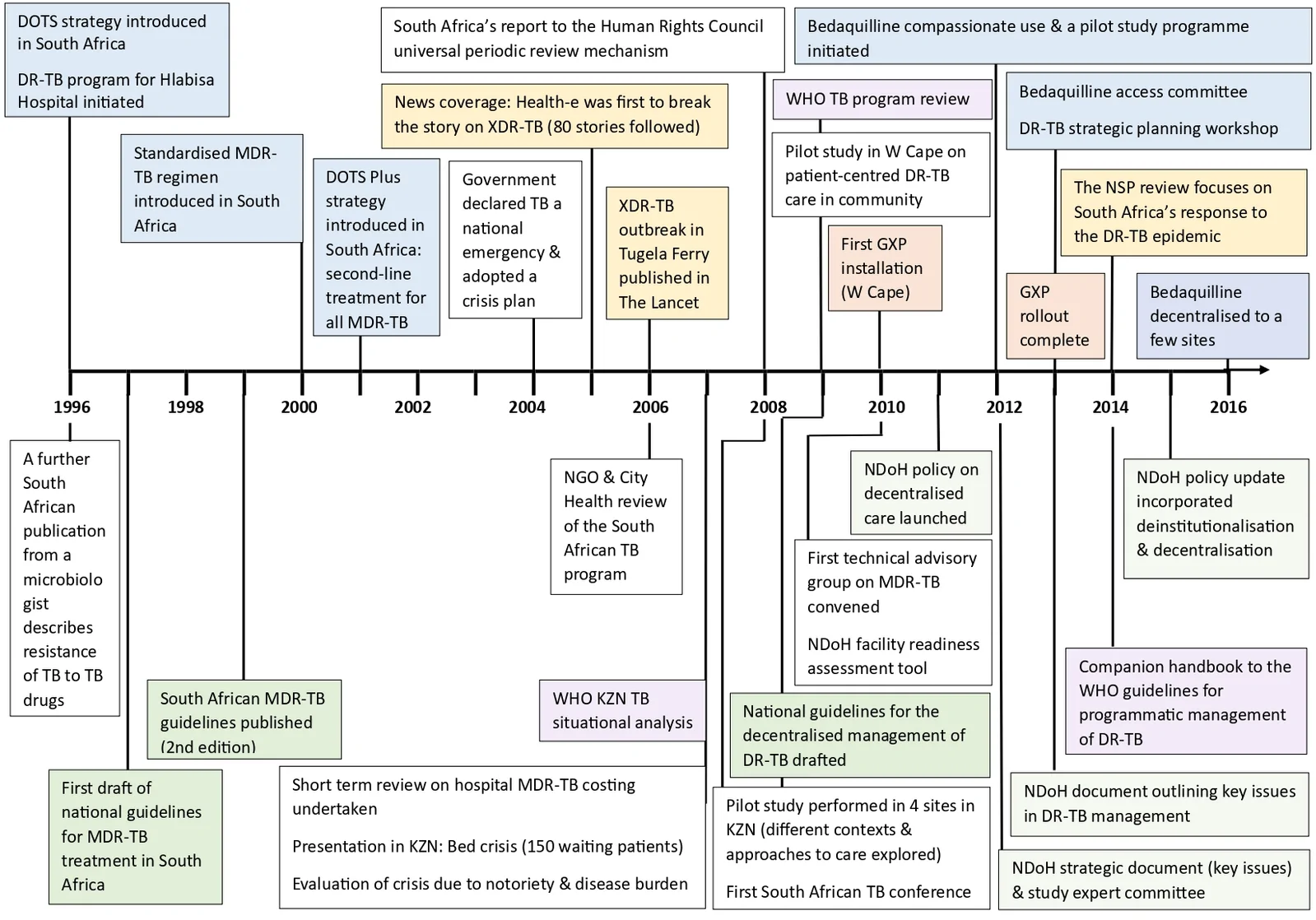
Timeline to indicate significant events impacting the DR-TB landscape in South Africa between 1996 and 2016. Yellow: media event; orange: laboratory event; blue: government intervention; purple: WHO input; green (dark): major government publication; green (light): minor government publication. DOTS, Directly Observed Treatment, Short-Course; DR-TB, drug-resistant tuberculosis; GXP, GeneXpert; KZN, KwaZulu-Natal; MDR-TB, multidrug-resistant tuberculosis; NDoH, National Department of Health (South Africa); NGO, non-governmental organisation; NSP, National Strategic Plan; TB, tuberculosis; XDR-TB, extensively drug-resistant TB.

### Theme 1: problem stream

Shortly after the XDR-TB outbreak in Tugela Ferry, in early 2007 reports emerged of excessively long waiting periods for admission and treatment initiation in specialised DR-TB hospitals. The problem was framed as a hospital bed crisis with limited health system capacity to manage the large number of people with DR-TB (figure 1). The team tasked with tackling the South African DR-TB problem was led by national TB directors, supported by provinces, particularly KwaZulu-Natal, given their experience in the Tugela Ferry outbreak, and international experts, including consultants from WHO. The NGO Médecins Sans Frontières (MSF) expanded their remit from HIV to include DR-TB in the Western Cape, advocating (along with others) for a change in South Africa’s policy on DR-TB management at national and international conference meetings, including the International AIDS Society Conference in 2009. It was not until 2010 that the ‘National Clinical Advisory Group’ on DR-TB was formed, just before the national policy on the decentralisation of DR-TB management was launched in 2011.

Key informants agreed that the national public health crisis of the Tugela Ferry XDR-TB outbreak was a ‘necessary evil’ that jolted into action many actors, including local, national and international TB experts.

What triggered it was maybe in 2006, the XDR outbreak in Church of Scotland [Tugela Ferry]. That really put TB back on the map…especially DR-TB (K001, researcher)

To assess the scale of the DR-TB problem in South Africa, a detailed situation analysis was conducted in KwaZulu-Natal by a task team led by the Department of Health, in collaboration with the WHO and the US Centers for Disease Control and Prevention. The analysis found a health system crisis of DR-TB bed capacity shortage in the context of the high number of people with DR-TB.

So, in one year you can treat 300 patients. However, in KwaZulu-Natal, in the lab [laboratory], there were 3000 MDR-TB patients that were diagnosed… (K005, global actor)

The situation analysis suggested that many people with DR-TB were waiting in the community to access DR-TB treatment and likely contributing to ongoing transmission. Attempts to increase bed capacity were made, but the scale of the problem was such that bed capacity expansion would have minimal impact. There was an added problem of ‘forced’ hospital admissions, where people with DR-TB had to stay in hospital, often against their will, for periods of up to 12 months.

Patients no longer wanted to be admitted. Other people just simply put it to us that they would rather die, they’re not going to go into hospital. We had really extreme and unexpected strong reactions towards being admitted*.* (K018, public sector implementer)

The policy of mandatory hospital admission was perceived to violate the rights of people with DR-TB who were often denied care when they refused hospital admission.

…now that is a stupid policy because it means you’re making me choose between caring for my three children and dying [by withholding life-saving treatment] …. I’m a salaried employee so if I had three months sick leave, I’d be fine. But many people are not salaried. I’ve got patients who refused admission for incredibly valid reasons. (K003, NGO actor)

The resulting circumstances precipitated a crisis. People with DR-TB would revolt, engage in protest action, disrupt hospital services, block the hospital entrance and at times jump the fence to escape from the hospital.^37^ The degree of force employed in the response to these protests was seen as violent and excessive. Security fencing, equated by key informants to that used in prisons, was erected at some hospitals and the police were often involved in responding to protests or returning people with DR-TB who had left the hospitals.

When we had a really strict centralised policy, there were times when the patients actually revolted. It’s happened in a number of hospitals*…* (K003, NGO actor)

Informants agreed that a punitive victim-blaming approach prevailed, with people considered responsible for their DR-TB because of self-inflicted poor adherence to previous TB treatment.

There is a very much punitive way of dealing with TB. ‘You’ve got drug resistant TB; it is your fault. You messed up the treatment and now you’re paying for it*.*’ (K018, public sector implementer)

The DR-TB programme, similar to DOTS, was seen to prioritise the activities and outcomes of the programme over the needs of people with DR-TB and to pay little attention to those who were yet to enter the programme. The ‘patient-blaming’ mentality was perceived to label and stigmatise people with DR-TB, compromise the therapeutic alliance and perpetuate the crisis of human rights.

*…*I mean more than half of our patients who get MDR-TB get it de novo, it’s a transmitted illness. So, they shouldn’t be punished, you know nobody should be punished for getting sick anyway*.* (K003, NGO actor).

### Theme 2: policy stream tensions

While it was already clear as early as 2006 that South Africa could not continue with an institutionalised care model for DR-TB, it would take another 5 years before the decentralisation policy was adopted. The policy actors who were against the shift in policy from centralised to decentralised care argued that an evidence base was needed to support the policy shift to decentralised care. A move away from a policy of institutionalised care was made more difficult because WHO, as the accepted global normative body, neither had a policy nor advocated for the decentralisation of DR-TB care.

When they conceptualised this concept of decentralisation, it was completely without evidence. In fact, a lot of things in TB are based on expert opinion. And with this, there was no expert opinion, it was just that—‘we have this backlog, people are dying, so what strategy can we employ*?’* (K001, researcher)

According to key informants who were part of the policy agenda-setting, there was initially much resistance towards decentralised care among TB experts, including from WHO. DR-TB care was understood to be a centralised service in South Africa, aligned with national and international WHO policies. Proposals of a decentralised service triggered fear among TB specialists of amplified drug resistance (ie, selection for resistance to additional anti-TB drugs, resulting from incorrect use of drugs or poor adherence to therapy), a fear with which some of the key informants could identify.

[Prominent DR-TB Clinical Expert] was very anti, very anti decentralisation because… for the simple reason… they felt that… if specialists didn’t manage DR-TB, it was going to amplify drug resistance…So I’ve been on the national drug-resistant advisory at the time when this (decentralisation) was being conceptualised. And I must say I was also sceptical about this*.* (K001, researcher)

The policy of centralised management of DR-TB recommended by WHO and the South African government was meant to ensure quality of care by providing specialised care by DR-TB specialists. Each province had a ‘centre of excellence’ for DR-TB, where people with DR-TB were admitted and managed.

WHO has a policy of centralised facility of managing drug-resistant TB because they perceive management of drug-resistant TB to be very specialised. And they felt that it was only specialists with experience that could manage drug-resistant TB. And also, incorrect management, incorrect drug treatment, or inappropriate use of drug treatment could potentially amplify resistance*.* (K001, researcher)

Key informants perceived the TB programme to have failed in detecting and acting on the emergence and growing frequency of DR-TB, which contributed to the XDR-TB crisis in Tugela Ferry. The Medical Research Council conducted a review of drug resistance surveys in four provinces in South Africa showing a reduction in the prevalence of resistance from 1965 to 1988 which was interpreted to indicate that resistance did not pose a threat.^38^

Late eighties we identified this problem…Ah at that time everywhere we went we were criticised, the Western Cape…we are wasting our time and money and all sorts of things, resources, with these drug-resistant patients because what we had to focus on was the normal TB programme*.* (K018, public sector implementer)

Compared with the rapid scale-up of the South African HIV programme, the DS-TB and DR-TB programmes were perceived to maintain old ways of practising healthcare. The philosophy of centralisation, admission and isolation, characterised in this study as a ‘control-based approach’, was seen to be very different from the philosophy of decentralised, widely accessible care observable in the HIV programme, characterised as an ‘access-based approach’. Key informants argued that the TB ‘control’ mentality hampered innovation in TB, human rights and access to care.

Of course, as you can imagine, most people that managed TB are very set in their ways. Unlike HIV, it’s a real old boys club, not just in South Africa, but globally*.* (K001, researcher)

Figure 3 illustrates how during the 5-year policy formulation period, tensions between two dominant schools of thought resulted in policy stagnation.

**Figure 3:**
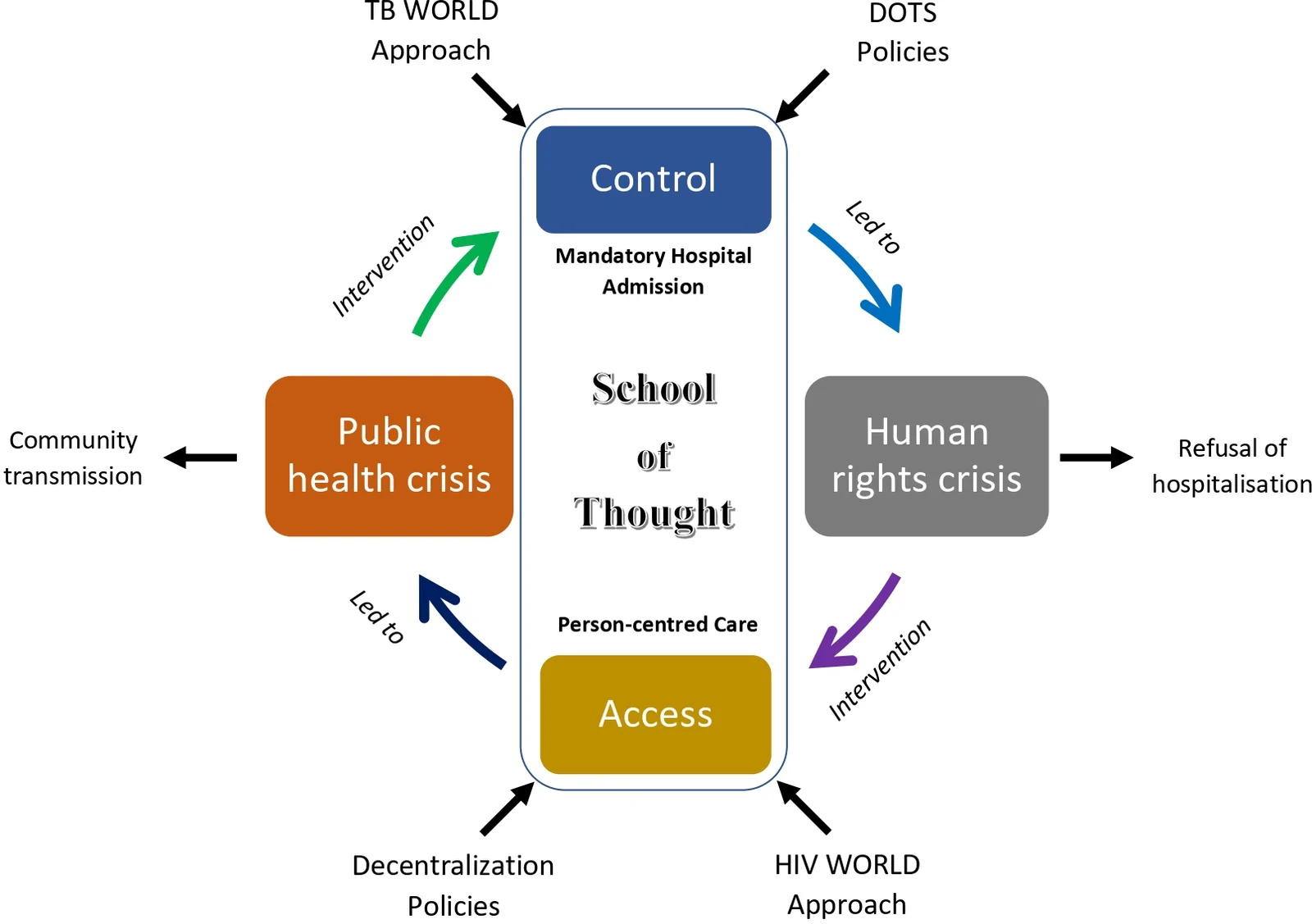
Tension between access-based and control-based schools of thought about decentralisation of drug-resistant tuberculosis care leading to policy stagnation. DOTS, Directly Observed Treatment, Short-Course.

### Theme 3: politics stream and the policy window

The policy process for the decentralised management of DR-TB in South Africa moved forward as a result of the distinct involvement of specific interest groups. The national task team, led by the TB directors in the National Department of Health, visited programmes in Peru in 2008 and Lesotho in 2011, where the NGO Partners in Health was already implementing decentralised DR-TB management. These visits were conducted for the purposes of benchmarking, given there was little scientific evidence available to support decentralisation.

So, the whole policy on decentralisation and community-based [care] were inspired by Partners in Health. They did a lot of work in the community….and they had to treat. They proved that actually what we’re doing in South Africa is wrong because our policy is to isolate. (K004, policy-maker)

As evidence from benchmarking visits began to influence the direction of policy debates, evidence was also shared from two South African pilot programmes, one in the Western Cape Province implemented by MSF, in collaboration with the City of Cape Town and the Provincial Department of Health, and another in KwaZulu-Natal Province implemented directly by the Department of Health and supported by academic partners. These decentralised pilot projects demonstrated feasibility and outcomes comparable to centralised programmes, indicating potential for a decentralised model.^39–41^ After extensive visits by the policy entrepreneurs to the pilot sites, it became clear that the Western Cape model was more suited to well-resourced urban or peri-urban settings, whereas the KwaZulu-Natal model could be emulated more widely across non-metropolitan areas in South Africa.

When we started engaging in this discussion, we picked up that there were two provinces in the country that were already doing some form of decentralised management. Two models really, two provinces inspired us from National to come up with a policy, KwaZulu-Natal and Western Cape*.* (K004, policy-maker)

Concurrently, research evidence emerged suggesting that early initiation of treatment for DR-TB might reduce mortality compared with treatment delayed by shortage of hospital beds, and was likely to reduce community transmission of DR-TB strains.^42^ These data supported arguments that it would be best to initiate DR-TB treatment among people awaiting admission to specialised TB hospitals, even if this was at non-specialised hospitals or clinics, and without admission. Therefore, the evidence to treat early reiterated the need for a decentralised DR-TB programme.

*…*but there was also research from [name of researcher noted] that actually the best way to cut transmission is to initiate effective treatment early. If we are able to diagnose early as well and initiate then you get better results*….* (K004, policy maker)

In 2009, a political shift took place, after the controversial national Minister of Health (widely considered an HIV denialist) was replaced, resulting in a transition from a health minister with a control-based school of thought to one with a more access-oriented approach considered friendly to policies from the ‘HIV world’.^43^

Policy actors who initially opposed the decentralisation policy later yielded largely because they were not able to offer better options, while the number of people with DR-TB continued to grow. The lessons from the benchmarking visits, pilot projects and emerging research provided enough impetus to bring about change in the national mood and willingness to implement the decentralisation policy. Most importantly, key informants believed that DR-TB was a public health problem that could be eradicated in South Africa, given the development of new drugs and using a more person-centred approach.

I think that if we could change the philosophy… from old-school to new-school, which is a respectful, scientific, evidence-based way of treating DR-TB then we could wipe DR-TB out of this country within 5 years. In South Africa, we can definitely wipe it out! (K003, NGO actor)

## Discussion

In this study, we observed that the policy change process leading to the launch of a national policy to decentralise DR-TB management in South Africa took place over 5 years (2006–2011). In accordance with Kingdon’s theory of agenda-setting (figure 1), the policy change was triggered by the perceived problem of a ‘crisis event’. In this case, several layers of crises emerged: the national TB crisis declared in 2004 was noted, but the 2006 Tugela Ferry XDR-TB outbreak triggered the DR-TB policy agenda-setting process. Immediately after the outbreak, studies revealed large numbers of untreated individuals living with DR-TB in the community, carrying the risk of ongoing disease transmission.^34 44–46^ National policy weaknesses resulted in failure to detect, monitor and respond to the emergence of DR-TB in the 1980s and 1990s, influenced largely by the policy position of the WHO through the DOTS strategy,^4^ which encouraged high TB prevalence countries to focus on DOTS and first-line TB treatment and did not prioritise drug-sensitivity testing and individualised treatment regimens for DR-TB. The underuse of sputum culture and sensitivity testing masked the growing DR-TB problem and contributed to DR-TB denialism, and partly explained the inaction prior to the Tugela Ferry crisis.^34^ The focus on ‘control’ in the DOTS-Plus strategy endorsed DR-TB clinical expertise being centralised in specialist hospitals, necessitating extended periods of mandatory hospitalisation, constrained by limited hospital bed capacity, with associated barriers of access for people to reach the centralised hospitals. In addition, a human rights crisis entered the policy change debates in 2008,^47^ driven largely by the mandatory hospital admission policy, presenting as violation of individuals’ freedom, negatively affecting their psychosocial and socioeconomic circumstances. Therefore, a combination of failures in surveillance, programmatic and policy responses in South Africa conspired in complex dynamic ways to create a conducive environment for a major DR-TB crisis.

During the period of policy stagnation (figure 3), we found that some experts espoused an approach to DR-TB consistent with the DOTS-Plus strategy^17^ and previously dominant at WHO, concerned about poor adherence to limited, costly drugs, the risk of amplification of resistance and limited capacity of non-specialists to manage a complex disease and use limited resources effectively. They called for mandatory hospitalisation throughout TB treatment, overseen by specialist clinicians, to ensure treatment completion, protect the few available drug options^17^ and prevent further transmission by isolation during treatment. This approach can be characterised as ‘control-based’, reflecting traditional thinking in the TB world. Other actors, concerned with the human rights crisis and access barriers, called for person-centred approaches through which individuals could receive care closer to their homes, retaining their autonomy and livelihoods, and avoid long waits for treatment in specialised hospitals.^48–51^ This can be characterised as an ‘access-based’ approach, more characteristic of the HIV world, where there was earlier emphasis on person-centred approaches and a human rights perspective on healthcare. The access-based school of thought considered that decentralised healthcare services closer to the homes of people with DR-TB could help them overcome access barriers and improve treatment adherence and retention in care.^41 50 52–54^ The conflict between these two schools of thought represents the emergent tensions between the world views of two major infectious disease programmes^55^ as shown in figure 3; the ‘HIV world’ which adopted more ‘access-based’ person-centred decentralised approaches, and the ‘TB world’ which retained the ‘control-based’ specialised centralised approaches. Development of a new policy was also hindered by the absence of explicit normative guidance from WHO.

In many low- and middle-income countries, decentralised healthcare services are provided by nurses in primary healthcare facilities,^56 57^ rather than specialised medical doctors. Therefore, advocacy for decentralised healthcare services could be perceived as at odds with the importance of high-quality services delivered by specialist clinicians, as DR-TB management was positioned by WHO. We found that these two schools of thought clashed during the policy agenda process, resulting in policy impasse (figure 3), while both acknowledged the problem and the need for a solution. At the time of policy formulation, the evidence base to support the decentralisation of DR-TB management was weak, and there was no explicit policy guidance from WHO for the decentralisation of DR-TB management. It was not until 2011 that, based mainly on advocacy and policy from South Africa, WHO issued a conditional recommendation to support ambulatory models of care not based principally on hospitalisation, on the basis of ‘very low-quality evidence’.^58^

The feasibility of the emerging models of decentralised DR-TB care outside specialist units in two South African provinces formed the evidence base which was presented to national government and TB experts to support the policy change. The change in South African political leadership in 2008 to a minister with a more access-oriented approach may have helped to reassure people opposed to decentralisation of DR-TB management, and it coincided with moves to adoption of new diagnostic technologies and drugs, such as Xpert MTB/RIF and bedaquiline, respectively.

### Limitations

The retrospective nature of the study carries a risk that informants did not perfectly recall key features of the policy agenda-setting process. However, document and literature reviews were used to corroborate the timeline of events. Further, cross-checking across transcripts, using a data matrix aligned with the framework approach, allowed us to assess consistency in the subthemes as they related to the identified trigger ‘problem’, policy tensions, and political policy ‘window’ as described above. Our interviews may not have included the full range of actors relevant to the policy process; in particular we have largely captured the experience of policy-makers and advocates, which may have underestimated the perspectives and importance of those driving policy from within lower tiers of the national TB programme.

## Conclusions

The South African DR-TB decentralisation policy was formulated in reaction to the health system’s inability to match limited specialised hospital bed capacity to the rapidly growing number of people in need of DR-TB management. Although policy scholarship identifies crises as important catalysts for agenda setting, the 5-year delay between recognition of the crisis (2006) and policy adoption (2011) illustrates how institutional inertia can persist even during a public health emergency. Our analysis suggests that two interrelated mechanisms impeded policy change. First, a dominant, centralised ‘control-based’ clinical paradigm, upheld by the specialist clinical community, prioritised containment through hospitalisation over patient autonomy because of concerns that decentralised care would accelerate the emergence and transmission of drug resistance. Second, South Africa’s close adherence to a cautious global normative framework meant that, in the absence of WHO guidance or an established evidence base for ambulatory DR-TB care, policymakers were reluctant to depart from prevailing international practice. Together, these factors created institutional gridlock in which professional risk aversion and defensive models of clinical governance outweighed growing evidence of community transmission and concerns regarding patients’ rights and access to care.

Policy change was ultimately catalysed not through top-down consensus, but through locally generated evidence from provincial pilot programmes, complemented by international benchmarking visits that demonstrated the feasibility of alternative models of care in settings where global guidance remained limited. South Africa’s experience suggests that overcoming policy stagnation during complex health emergencies requires more than accumulating evidence of system failure. It also requires challenging entrenched institutional gatekeeping, creating space for locally generated innovation, and moving beyond defensive, control-oriented models of care before structural failure makes reform unavoidable.

## Supplementary material

10.1136/bmjopen-2025-115476 online supplemental file 1

## Data Availability

No data are available.
