# Supplementary material for: “We were caught sleeping”: formulation of national policy to decentralise management of drug-resistant tuberculosis in South Africa – a qualitative policy analysis study
Source: BMJ Open. 2026 Sep 28;16(9):e115476. doi: 10.1136/bmjopen-2025-115476 (PMC13630063; doi:10.1136/bmjopen-2025-115476)
Supplement: Supplementary file 1 [file bmjopen-2025-115476.reviewer_comments.pdf]

PEER REVIEW HISTORY

BMJ Open publishes all reviews undertaken for accepted manuscripts. Reviewers are asked to complete a checklist review form and are provided with free text boxes to elaborate on their assessment. These free text comments are reproduced below.

ARTICLE DETAILS

Title (Provisional)

“We were caught sleeping”—formulation of national policy to decentralise management of drug-resistant tuberculosis in South Africa: a qualitative policy analysis study

Authors

Moshabela, Mosa; Dickson, Lindy; Jassat, Waasila; Cox, Helen; Kielmann, Karina; Loveday, Marian; Black, John; Mlisana, Koleka; Nicol, Mark; Grant, Alison

---

VERSION 1 - REVIEW

**Reviewer**	**1**
**Name**	**Andersen, Åse Bengård**
**Affiliation**	**Rigshospitalet, Infectious Diseases**
**Date**	**10-Feb-2026**
**COI**	**None**

”We were caught sleeping” is the catching title of this manuscript trying to analyze and pin-point factors important for the management of Drug Resistant (DR) TB in South Africa (SA).

The world experiences epidemics with unpredictable intervals: as examples: influenza, Ebola and lately COVID was challenging the health systems in many parts of the world. We are still reflecting on the covid response to understand how we responded and what could be improved for “next time”.

TB has more or less always been on the agenda, and at times “promoted” to a global health emergency but somehow the response pattern – especially in hindsight – does appear slow and inefficient.

It is well known that SA was one of the hardest hit regions in the world with respect to DR and later XDR TB, so an analysis of “the system-response" to this crisis is very interesting and relevant for health-care staff and politicians involved in management of epidemics.

The study period was limited to the period between 2004 to 2016, and the methodology is thoroughly described in the manuscript. A substantial number of stake holders were inter-

viewed during 2018 to 2019 to decipher the events eventually leading to a change in national policy.

The observation period ended right at the time when oral and shorter regimens became avail-able.

• Was this cutting point chosen for that reason?

• Did the new regimens influence the decentralization process?

SA was in a terrible situation and had by no means capacity to treat the increasing number DR TB cases (300 beds vs 3000 pts) a situation nowadays designated “a burning platform”.

This is clear in retrospect – but why didn’t anyone conclude that at the time?

The “system” was divided into two groups: one trying to reinforce the existing strategy: re-sistance develops due to non-adherence i.e. it’s the patient’s fault opposed to newer views supported by promising results from pilot studies and later also results from Peru and Lesotho.

The WHO is reproached for responding reluctantly and being out of “sync” with real life conditions for a long time. But still it is hard to understand why it was so difficult to realize that “the boat was sinking”? (I guess this is what is termed “the tipping point” in the paper.) Who was asleep?

The authors identify a lot of important factors – and this is clearly a huge topic. However, I do find the conclusions somewhat vague (e.g. …seek healthcare solutions that balance quality with accessibility…)

I suggest that the authors strengthen their conclusions and reveal their opinions on which mechanisms (out of the many) played a major role in slowing the decentralization process.

In conclusion a well written and interesting paper - only minor revisions suggested.

Reviewer **2**

Name **Mitchell, Ellen**

Affiliation **Mycobacterial Diseases and Neglected Tropical Diseases Unit, Department of Public Health, Institute of Tropical Medicine, Antwerp, Mycobacterial Diseases and Neglected Tropical Diseases Unit**

Date **16-Feb-2026**

COI **None**

There are few oral histories of this important period in DR-TB policy and few attempts to unpack what happened. Most of the peer review literature is self-congratulatory or donor-driven, so it is useful to apply a lens to the policy process that led to the gradual transition to DR-TB ambulatory care.

Although the topic is important and the methods make sense, the execution of the discourse analysis has a number of minor and more substantive shortcomings that hinder its overall value for the field. A deeper dive is warranted.

Major Comments:

1. Despite DR-TB diagnosis and treatment being a rather complex and evolving field, the authors have presented it as a rather binary "good" versus "evil" gloss. The richness and nuance of the interviews is whittled down to a "human rights" versus "adherence" story. To boil it down to two competing camps with a single axis of "control" versus" access" does a over-distillation of a very complex picture. Did the ethnographic and policy information not present any other valid dilemmas or considerations that stakeholders had to balance? Is the old stereotype about boring, staid TB and innovative HIV really the analytic take home?

2. Missing from the manuscript is information on the scale up of access to DST over time, the periodicity and infrastructure of clinical DR-TB monitoring for drug toxicities, the shifting risks of toxicity of the regimens, issues of liability, the role of donors in provision of specific drugs as donations, the role of staffing at DR-TB hospitals.

3. Although a review of policy documents is included in the manuscript there is very little reference to the text of these documents. It would be complimentary if data from the policy documents were offered to buttress the many claims. What is not said in these documents? What are the gaps in the early guidelines? How do they embody violence and control?

4. The story is a very condensed and leaves of the names of many of the people and institutions that were influential. Invisibilizing the key actors and only occasionally mentioning mission-driven NGO seems as if this were unimportant --but the history of the ideas, missions, influence ecosystems, networks, relationships are as critical to this story as any guidelines. The cross-fertilization of the various stakeholders remains a black box, despite the promise of an analysis of politics. Even attempts to influence policy makers by sending them on study trips abroad is described innocuously and confusingly as "benchmarking". This depersonalized version is quite opaque, but then it is even further abstracted by use of concepts like " the national mood" which make the agency of individual players quite amorphous.

5. Tell us move about this "control" ideology. Is this "control" of patients or drugs? How does this DR-TB hospitalization debate relate to the whole philosophical debate about direct observation of Tx? If Dr-TB Tx is moved to ambulatory but still under DOT, is that emancipatory or 'dignified' or just shifting the cost of care to the patient (catastrophic costs)?

Minor comments

1. The study rationale is not yet compelling.

2. The study is described as cross-sectional but it is more like an oral history to me in that it probes the recollections.

3. The discourse analysis methods are incompletely described – Please provide more detail on what was done with the transcripts. How were the codes developed and applied? What categories were used? How many coders were there? and what efforts were made to ensure inter-rater reliability?

4. The reflexivity statement is not transparent, let alone reflexive, about the authors' vital contributions to RSA TB policy. The authors are hardly passive observers in the processes they describe.

5. The manuscript would benefit from more nuance in figure 2 - it seems too binary and stark - conceptualize all the forces and how they pushed and pulled at the policies.

6. The timeline is very helpful – It might be even better if it focused narrowly on the study period and had policy change on the same axis with regimen change, ambulatory /hospitalization, and some sort of outcomes.

7. The interviewees report some "tensions" as though this were a philosophical difference. But the authors seem to suggest that decentralization was also a consequence of the collapse of the old system due to lack of capacity. There weren't enough beds and people were not being care for fast enough. So there was not really any choice. That argument stands in contrast with the statement that they moved deliberately to an "evidence-based" , patient-centred" model as a consequence of principled advocacy. Did the old paradigm implode and this decentralization thing was a stop gap or a contingency? These two explanations seem hard to reconcile and the authors could do more to help readers to better understand the meanings and framings behind these competing narratives.

8. The advent of all oral regimens and the rise of BDQ resistance is not mentioned, which is a choice that could be questioned.

This is an important manuscript, especially if it can be further grounded in the insights from the interviews and the discourses can be more critically analyzed to help readers to understand how and why the policy changed.

VERSION 1 - AUTHOR RESPONSE

Reviewer: 1
Dr. Åse Bengård Andersen, Rigshospitalet
Comments to the Authors:
"We were caught sleeping" is the catching title of this manuscript trying to analyze and pin-point factors important for the management of Drug Resistant (DR) TB in South Africa (SA).

The world experiences epidemics with unpredictable intervals: as examples: influenza, Ebola and lately COVID was challenging the health systems in many parts of the world. We are still reflecting on the covid response to understand how we responded and what

could be improved for “next time”.

TB has more or less always been on the agenda, and at times “promoted” to a global health emergency but somehow the response pattern – especially in hindsight – does appear slow and inefficient.

It is well known that SA was one of the hardest hit regions in the world with respect to DR and later XDR TB, so an analysis of “the system-response" to this crisis is very interesting and relevant for health-care staff and politicians involved in management of epidemics.

The study period was limited to the period between 2004 to 2016, and the methodology is thoroughly described in the manuscript. A substantial number of stake holders were inter-viewed during 2018 to 2019 to decipher the events eventually leading to a change in national policy.

The observation period ended right at the time when oral and shorter regimens became avail-able.
• Was this cutting point chosen for that reason?

The work was part of a larger funded research programme exploring care pathways for people with DR-TB which ran between 2016 and 2020. The interviews for this policy analysis were done early in the programme (2016-2017) because they provided important context for other components of the programme.

• Did the new regimens influence the decentralization process?
The process of policy formulation (2006-2011) precedes the introduction of new regimens by some years. All-oral regimens started to be used programmatically in South Africa in 2018 and the earliest WHO guidance on all-oral regimens was published in 2022 and so did not influence the process described in this paper.

SA was in a terrible situation and had by no means capacity to treat the increasing number DR TB cases (300 beds vs 3000 pts) a situation nowadays designated “a burning platform”.

This is clear in retrospect – but why didn’t anyone conclude that at the time?

Clinical staff working in DR-TB in South Africa were very well aware of the escalating crisis, as our results illustrate. The problem was that national policy on how to deliver TB care was (and still is) primarily led by normative guidance from WHO, and during the period we describe WHO had no recommendations for how decentralised care for

people with DR-TB should be organised. As we describe, local and national experts had differing perspectives on how DR-TB should be managed, with some feeling strongly that patients should be hospitalised for initial treatment for the reasons we describe, even though that was clearly not working. Thus national policy had to be developed de novo*, which was unusual, and contributed to the slow progress with developing the policy. As above, we have added a sentence to the discussion to emphasise this point (lines 454-455).*

The “system” was divided into two groups: one trying to reinforce the existing strategy: resistance develops due to non-adherence i.e. it’s the patient’s fault opposed to newer views supported by promising results from pilot studies and later also results from Peru and Lesotho.

The WHO is reproached for responding reluctantly and being out of “sync” with real life conditions for a long time. But still it is hard to understand why it was so difficult to realize that “the boat was sinking”? (I guess this is what is termed “the tipping point” in the paper.) Who was asleep?

Historically, South Africa’s capacity to deploy an immediate programmatic response to the escalating pressures of DR-TB was constrained by reliance on international normative guidance. Standard public health protocol dictated awaiting formal policy frameworks from WHO; however, at the time, no global guidelines or operational policies existed regarding the decentralization or deinstitutionalization of DR-TB care. Consequently, the country found itself in uncharted public health territory, facing an acute bed shortage and rising transmission that could not wait for international consensus. Forced to operate ahead of global policy, South African authorities and local implementation partners had to independently generate the necessary clinical and operational evidence. Through localized pilot projects—most notably in high-burden sub-districts like Khayelitsha—the country successfully demonstrated the viability of community-based and nurse-initiated care. This pioneering local evidence base was subsequently used to design, pilot, and formally launch the historic 2011 National Policy Framework on Decentralised and Deinstitutionalised Management of MDR-TB, effectively establishing a precedent for decentralized care years before it was formally integrated into global WHO recommendations. As above, we have emphasised this point by including it in the abstract (line 5) and adding a sentence to the discussion (lines 454-455).

The authors identify a lot of important factors – and this is clearly a huge topic. However, I do find the conclusions somewhat vague (e.g. …seek healthcare solutions that balance quality with accessibility…)

I suggest that the authors strengthen their conclusions and reveal their opinions on which mechanisms (out of the many) played a major role in slowing the decentralization process.
We appreciate the reviewer's recommendation to strengthen the conclusion by moving away from general advice and explicitly naming the primary mechanisms that stalled policy progress. We have extensively revised the Conclusion (lines 485-507) to address this point. Specifically, we argue that the primary mechanism delaying policy formulation for five years was the deep-seated "control-based" institutional ideology among clinical elite networks, heavily reinforced by the lack of global normative guidance from the WHO. This created a profound systemic inertia driven by a collective professional fear of amplifying drug resistance, which prevailed over urgent patient access and human rights considerations until systemic collapse left no other option.

In conclusion a well written and interesting paper - only minor revisions suggested.

** **

Reviewer: 2
Dr. Ellen Mitchell, Mycobacterial Diseases and Neglected Tropical Diseases Unit, Department of Public Health, Institute of Tropical Medicine, Antwerp
Comments to the Authors:
There are few oral histories of this important period in DR-TB policy and few attempts to unpack what happened. Most of the peer review literature is self-congratulatory or donor-driven, so it is useful to apply a lens to the policy process that led to the gradual transition to DR-TB ambulatory care.

Although the topic is important and the methods make sense, the execution of the discourse analysis has a number of minor and more substantive shortcomings that hinder its overall value for the field. A deeper dive is warranted.

Major Comments:
1. Despite DR-TB diagnosis and treatment being a rather complex and evolving field, the authors have presented it as a rather binary "good" versus "evil" gloss. The richness and nuance of the interviews is whittled down to a "human rights" versus "adherence" story. To boil it down to two competing camps with a single axis of "control" versus" access" does a over-distillation of a very complex picture. Did the ethnographic and policy information not present any other valid dilemmas or considerations that stakeholders had to balance? Is the old stereotype about boring, staid TB and innovative HIV really the analytic take home?
We agree that this is a complex picture. Our analysis suggested that "control" versus

"access" was a dominant tension between actors opposing versus supporting decentralisation, and key informants specifically contrasted the difference in approach between the "old boys' club" of TB leadership compared to HIV (see lines 359-360) and "old-school to new-school" (line 407). However there were of course other dilemmas as illustrated in figure 2 under "policy agenda". "Control" is a useful shorthand but covered many components as we summarise in the discussion (lines 426-429, 435-443).

2. Missing from the manuscript is information on the scale up of access to DST over time, the periodicity and infrastructure of clinical DR-TB monitoring for drug toxicities, the shifting risks of toxicity of the regimens, issues of liability, the role of donors in provision of specific drugs as donations, the role of staffing at DR-TB hospitals.

We agree with the reviewer that these are important related issues, but they are not central to the aim of the paper to describe the process of policy formulation. Information about key events such as the introduction of NAAT testing for TB is included in the timeline (figure 1). Throughout the calendar period that we describe in this paper (2006-2011) the standard treatment regimens for DR-TB were based on the requirement for an injectable agent for at least six months. Bedaquiline started to be used in pilot studies in 2012, as shown in the timeline, and all-oral regimens started to be used programmatically in 2018. Some issues about drug toxicity were raised in the interviews and are included in the results, but are not the focus of the paper.

3. Although a review of policy documents is included in the manuscript there is very little reference to the text of these documents. It would be complimentary if data from the policy documents were offered to buttress the many claims. What is not said in these documents? What are the gaps in the early guidelines? How do they embody violence and control?
As described earlier, the purpose of the document review was to create a timeline/ chronology of events rather than to generate material for analysis. We have revised the methods section on document review (lines 136-146) to clarify this point.

4. The story is a very condensed and leaves of the names of many of the people and institutions that were influential. Invisibilizing the key actors and only occasionally mentioning mission-driven NGO seems as if this were unimportant --but the history of the ideas, missions, influence ecosystems, networks, relationships are as critical to this story as any guidelines. The cross-fertilization of the various stakeholders remains a black box, despite the promise of an analysis of politics. Even attempts to influence policy makers by sending them on study trips abroad is described innocuously and confusingly as "benchmarking". This depersonalized version is quite opaque, but then it

is even further abstracted by use of concepts like “ the national mood” which make the agency of individual players quite amorphous.

We have tried to balance providing an informative description of policy formulation with our ethical imperative to protect the anonymity of our key informants, many of whom were influential in the process (whether in favour of or against decentralised care). As mentioned in earlier responses, we have added a categorisation of interviewee to each quote. We name prominent institutional bodies, such as the World Health Organization (in multiple places), MSF (line 262 and elsewhere), the South African National Department of health (line 276 and elsewhere), the US Centers for Disease Control and Prevention (line 275), Partners in Health (line 368 and elsewhere), and the National Clinical Advisory Group (line 265). We describe “cross-fertilisation” as described in the interviews, for example visits to Partners in Health decentralisation projects. We do not think that visits to DR-TB decentralisation projects outside South Africa was an “attempt to influence” policy makers but rather a way for policy-makers to see for themselves examples of what was done elsewhere in order to inform the policy process, as described in lines 365–370.

5. Tell us move about this "control" ideology. Is this "control" of patients or drugs? How does this DR-TB hospitalization debate relate to the whole philosophical debate about direct observation of Tx? If Dr-TB Tx is moved to ambulatory but still under DOT, is that emancipatory or 'dignified' or just shifting the cost of care to the patient (catastrophic costs)?

As above, “control” pertained to several things including control of patients, and of drugs, and of inpatient facilities, as summarised in the discussion (lines 435-443). We agree that there is probably a relationship between attitudes to decentralisation of DR-TB care and to direct observation of therapy, as we mention in the results on lines 328-329, but direct observation of therapy (where this implies “supervised swallowing”) is a vast topic which we did not explore in this study. We agree that decentralisation may have shifted some costs to patients, but ambulatory care does not necessarily mean a large increase in transport costs for patients. In many clinics in South Africa “direct observation” of therapy, where that means that a patient has to attend the clinic daily to receive their TB treatment, has not been practiced for some years, and patients rather attend their clinic once a month to collect their treatment. In some rural areas, before the advent of all-oral regimens, care for people with DR-TB involved “injection teams” doing home visits multiple times per week to give the injectable agents, and in this case the health system continued to bear the cost of delivery of care.

Minor comments

1. The study rationale is not yet compelling.

We responded to this point earlier. A particular rationale is that it is unusual for countries to change policy on the management of a major disease when this has not been set out in normative guidance from WHO. In this case, WHO had not provided such guidance. We have edited the abstract (line 5) and the introduction (lines 94-95) to make this clear:

"In response, deliberations on how to manage the crisis led to the beginning of a shift in national policy, resulting in a policy to decentralise services for DR-TB in 2011; at this time there was no normative guidance from WHO concerning decentralisation of DR-TB care."

2. The study is described as cross-sectional but it is more like an oral history to me in that it probes the recollections.

We agree that "cross-sectional" is not a good description of the study design. It is better described as a qualitative policy analysis. We have edited the study design section of the methods accordingly (line 109).

3. The discourse analysis methods are incompletely described – Please provide more detail on what was done with the transcripts. How were the codes developed and applied? What categories were used? How many coders were there? and what efforts were made to ensure inter-rater reliability?

The interview transcripts were analysed using a framework approach whereby the main themes aligned to the streams in Kingdon's framework (i.e. problem stream, policy stream and politics stream). Within these macro-themes, open thematic coding was used inductively to generate sub-codes which are listed in figure 2. Coding was done by MM. We have edited the data analysis section to explain this more clearly (lines 195 – 202):

"We used a directed thematic analysis approach informed by Kingdon's Multiple Streams Framework[30] to analyse the primary qualitative data from key informant interview transcripts. This framework provided our a priori operational codebook, sorting data from transcripts into three macro-themes: problem stream, policy stream and politics stream. Within these macro-themes, open thematic coding was executed inductively to generate descriptive sub-codes as shown in Figure 2 (e.g. "mandatory admissions", "DOTS-Plus strategy", "resistance vs. innovation"). "

4. The reflexivity statement is not transparent, let alone reflexive, about the authors' vital contributions to RSA TB policy. The authors are hardly passive observers in the processes they describe.

The detailed reflexivity statement provided as a supplementary appendix was a requirement for BMJ Global Health but is not required by BMJ Open, so we have removed it. Some of the authors had a role in the process of policy formulation, but they were not involved in data collection. A sentence has been added to the paragraph on research team and reflexivity (lines 133-134) which reads:
"Some of the authors of this paper (not including MM and WJ who did the interviews) had roles in policy formulation."

5. The manuscript would benefit from more nuance in figure 2 - it seems too binary and stark - conceptualize all the forces and how they pushed and pulled at the policies.

Figure 1 (previously figure 2) illustrates both the highest-level codes (equivalent to the "streams" in Kingdon's framework) and the subcodes which emerged from data analysis, and how these came together in formulation of policy (column 3 and 4). We think it works better to keep this figure simple to show the coding framework.

6. The timeline is very helpful – It might be even better if it focused narrowly on the study period and had policy change on the same axis with regimen change, ambulatory /hospitalization, and some sort of outcomes.

Thank you. We think that there is value in the timeline extending beyond the period 2006-2011 to give the reader more contextual information about the evolution of DR-TB policy in South Africa. This figure is already quite busy and it would lose clarity if we added more information.

7. The interviewees report some "tensions" as though this were a philosophical difference. But the authors seem to suggest that decentralization was also a consequence of the collapse of the old system due to lack of capacity. There weren't enough beds and people were not being care for fast enough. So there was not really any choice. That argument stands in contrast with the statement that they moved deliberately to an "evidence-based" , patient-centred" model as a consequence of principled advocacy. Did the old paradigm implode and this decentralization thing was a stop gap or a contingency? These two explanations seem hard to reconcile and the authors could do more to help readers to better understand the meanings and framings behind these competing narratives.

We believe that both these explanations are correct and were happening simultaneously. It became increasingly clear that the old system requiring prolonged hospitalization was failing. As we describe, some actors were adamant that treatment should remain centralised while others advocated for decentralisation, but in the absence of normative guidance for how this should be done, there was no blueprint to follow, which made policy development much more difficult. As we describe, some clinical "entrepreneurs" experimented with different ways of delivering care which were

effectively pilot projects from which others could draw lessons on possible models of care.

8. The advent of all oral regimens and the rise of BDQ resistance is not mentioned, which is a choice that could be questioned.

All-oral regimens started to be rolled out in South Africa in 2018 which post-dates the period under examination (2006-2011) and the interviews and so we have not included discussion of this or of bedaquiline resistance since neither were influences on the process.

This is an important manuscript, especially if it can be further grounded in the insights from the interviews and the discourses can be more critically analyzed to help readers to understand how and why the policy changed.

VERSION 2 - REVIEW

Reviewer 2

Name Mitchell, Ellen

Affiliation Mycobacterial Diseases and Neglected Tropical Diseases Unit, Department of Public Health, Institute of Tropical Medicine, Antwerp, Mycobacterial Diseases and Neglected Tropical Diseases Unit

Date 01-Sep-2026

COI

The authors have tightened up the methods, and sharpened the claims, adding some nuance, while leaving most of the manuscript as it was - with the central claims, ideas, figures, and context as before. Even though I think this manuscript could have been better with more reflexivity and a wider lens, it is still pretty useful as is. It characterizes a specific point of view and moment in time. The fact that it was written in 2026 when we are now dealing with the consequences of these choices and events, is still pertinent, even if the authors choose not to acknowledge what came after. As a snapshot, the paper works. The authors have cited the need to protect the confidentiality of participants as the reason for keeping the nitty gritty of policy reform a bit vague. I can appreciate this, but I wonder if it helps us. In any event, the paper will be good for sparking debate. Even if I still feel that the sides are drawn too starkly, this text may be useful for catalyzing others to write about this period and to bring in other sides to this complex and important story.
