# Supplementary material for: “We were caught sleeping”: formulation of national policy to decentralise management of drug-resistant tuberculosis in South Africa – a qualitative policy analysis study
Source: BMJ Open. 2026 Sep 28;16(9):e115476. doi: 10.1136/bmjopen-2025-115476 (PMC13630063; doi:10.1136/bmjopen-2025-115476)
Supplement: Supplementary file 2 [file bmjopen-16-9-s001.docx]

**Moshabela et al.: “We were caught sleeping”**

**Interview topic guide**

*Explanations and prompts are provided in blue.*

| Date |  |
| --- | --- |
| Province |  |
| Researcher name |  |
| Unique identifier code 1 for primary interviewee |  |

**POLICY FORMULATION:**

1. What do you think motivated the idea to decentralise DR-TB in SA?

2. In your opinion, what were the main objectives intended by the decentralisation of DR-TB policy?

3. What issues and considerations necessitated and informed the design of the policy for decentralisation of DR-TB?

- *Epidemiological e.g. disease burden, transmission*
- *Economic, cost-effectiveness*
- *Ethics and human rights questions*

4. What were the expected outcomes in implementing the policy?

5. What were the steps followed in planning and designing the policy?

6. Who was consulted to develop the policy?

7. Who advised and assisted with the policy costing, funding allocation and financing flows which were linked to the decentralisation policy?

- *Additional monies / sources invested?*

8. Who are the main role players involved in the policy design?

- *Inclusion of correctional services, SASSA, immigration, prehospital transport?*

9. Was there senior support at different level of the health care system (national, provincial and district) for the decentralisation of DR-TB policy?

- *Relative priority/value accorded to DR-TB*

10. Were any other levels of government involved in the decentralization process?

11. How was harmonization of the decentralization process facilitated?

12. Were there any dynamics between the national and provincial expectation for decentralisation?

13. What is needed to ensure success of policy formulation?

- *Situating DR-TB within broader health care reforms in SA*

**POLICY IMPLEMENTATION:**

14. Is there an implementation (operational) plan?

15. Who are the main contributors, implementers, decision-makers, players and partners to implementation?

- *Meanings of decentralization – rationale, scope, models of care*
- *Role of private sector management of DR TB and relationship to public sector*
- *Organisation of decentralized care - governance structures*
- *Devolution of authority and decision-making in clinical care and expertise*

16. How has the implementation unfolded this far in your setting – reconstruct timelines, milestones, and progress?

- *Historical timeline – key events and actors*

17. What has been done to ensure that the implementation of decentralisation objectives are met?

- *What are the drivers, facilitators and barriers to implementation?*

18. Can you provide examples of any special or vulnerable population groups taken into by the policy or its implementation?

- *Key populations (hard-to-reach, paediatric, prisoner, psychiatric, XDR failures, immigrant)*

19. What has been the role of Xpert testing or other diagnostics in decentralisation?

20. How has the Bedaquilline introduction / rollout been incorporated into the decentralisation strategy?

21. What are the different categories of staff involved in the implementation of the DR-TB policy – how are they organised and trained?

- *Staffing and staffing arrangements – designated staff for DR TB*
- *Training, mentoring, and supervision of health workers*
- *Health worker responses e.g. nurses – unintended consequences*
- *Role of community health workers – barriers/enablers to their engagement e.g. issues of compensation, management, supervision, staff turnover, patient confidentiality.*

22. How does this decentralisation introduction and strategy affect the patients?

23. Do you think that decentralisation has had an impact on patient reliability or retention in care?

24. What other resources have been necessary to ensure the successful implementation of the policy?

- *Drug procurement services*
- *SASSA involvement*

25. Describe the financing flows that are in place in order to ensure the successful implementation of the policy?

26. Does language use, ie: certain labels and terms affect this process of decentralisation?

- Eg: treatment failures, defaulters, TDR, XDR

27. Are there any issues not taken into account during the decentralisation focus?

- eg: infection control?

28. ‘Are any special circumstances around DR-TB patients taken into account during implementation?

- *Disability grant in relation to DR TB patients*
- *Nutritional support programme for DR TB patients*
- *Roll-out of new regimens*
- *Flexibility in adapting services and guidelines according to patient needs/demands*
- *DR TB learners at schools / in active public service (including health care workers, teachers, etc)*
- *Failed XDR clients (palliative care / family infection control, community support)*
- *New drugs coming into use which could involve previous ‘treatment failure patient’ recall?*
- *Prisoner discharge transfers and parole follow-up / tracing*
- *Key populations’ (hard-to-reach, paediatric, prisoner, psychiatric, XDR failures, immigrant)*

29. Has the WHO evaluation had any influence in decentralisation of DR-TB?

30. How do targets play a role in decentralisation? Eg: NSP targets

31. Which NGO funded stimulated / projects have contributed to / impacted upon this process?

- Eg: Jhpiego

32. What factors do you consider likely to hinder the success of the policy implementation?

- *Legal framework e.g. duty to divulge diagnosis*
- *Political agendas*
- *TB registry reporting*
- *Financial allocation*
- *Pharmaceutical restrictions*
- *Training, mentoring & supervision of staff*
- *Patient resources & transport access*
- *location/environment/catchment area*

33. What are your comments about this process being an ‘unfunded mandate’?

34. Would you say that you were generally supportive or not supportive of the program to implement the national decentralisation plan?

35. What do you think is needed to ensure success of policy implementation?

- *Situating DR-TB within broader health care reforms in SA*

**HEALTH SYSTEM COMPONENTS:**

36. Can you please describe how communication takes place between facilities?

- *Communication mechanisms across facilities, resources for this (email, telephone, fax, formal stationary), who’s responsibility.*

37. What are the working arrangements in the current decentralisation system?

- *spatial arrangement*
- *staff hours in relation to patient load*
- *staff workload in relation to other services (that may or may not be integrated)*
- *remuneration*
- *transport issues*
- *training, mentoring & supervision*
- *infection control (staff, other patients & index patient)*

38. In what way have policy directives been adapted to become context-specific models of care? What influences these models of care?

- *location/environment/catchment area*
- *Patient age, comorbidities (eg: HIV status)*
- *Use of laboratory resources / expertise*
- *Resources (?patient?)*
- *burden of disease*
- *local expertise*
- *role of NGOs, advocacy groups, and external donor funded initiatives*
- *emergence of ‘champions’*

39. Can you describe the processes of facility reporting systems for DR-TB – give challenges?

- *Availability and barriers associated with existing facility reporting systems*
- *Fragmentation of information*
- *Getting patients on to the electronic registration systems (what categories of patients are included/excluded and why?)*
- *Relationship between EDR and ETR & private laboratories & NHLS? different data sources*
- *Non-notification by PP of DR TB cases*
- *Paediatric cases*
- *Prisoners*
- *Immigrants*

40. What are the governance systems in the current decentralisation system?

- *Integration of DR TB services within existing health and social care systems*
- *Role of private sector management of DR TB and relationship to public sector*
- *Organisation of decentralized care - governance structures*
- *Devolution of authority and decision-making in clinical care and expertise*
- *Accountability vis-à-vis reporting*
- *Operationalising the policy in the form of protocols, guidelines – distribution at what levels*
- *Anticipated risks versus challenges faced in implementation*

41. What is your view of the staffing make up utilized in a decentralized model?

42. How is quality control ensured?

- *Auditing and quality control*

43. What lessons have been learned thus far with regards to the decentralisation process?

44. What is needed to ensure success with decentralisation?

45. Do you have any other comments or contributions with regards to decentralisation?

**GENERAL:**

46. Can you tell us in your own words what is meant by decentralisation of DR-TB?
